# Current Status of Angiogenic Cell Therapy and Related Strategies Applied in Critical Limb Ischemia

**DOI:** 10.3390/ijms22052335

**Published:** 2021-02-26

**Authors:** Lucía Beltrán-Camacho, Marta Rojas-Torres, Mᵃ Carmen Durán-Ruiz

**Affiliations:** 1 Biomedicine, Biotechnology and Public Health Department, Cádiz University, 11519 Cadiz, Spain; lucia.beltrancamacho@alum.uca.es (L.B.-C.); marta.rojas@uca.es (M.R.-T.); 2 Institute of Research and Innovation in Biomedical Sciences of Cadiz (INIBICA), 11009 Cádiz, Spain

**Keywords:** critical limb ischemia, neovascularization, angiogenesis, arteriogenesis, cell therapy, secretomes

## Abstract

Critical limb ischemia (CLI) constitutes the most severe form of peripheral arterial disease (PAD), it is characterized by progressive blockade of arterial vessels, commonly correlated to atherosclerosis. Currently, revascularization strategies (bypass grafting, angioplasty) remain the first option for CLI patients, although less than 45% of them are eligible for surgical intervention mainly due to associated comorbidities. Moreover, patients usually require amputation in the short-term. Angiogenic cell therapy has arisen as a promising alternative for these “no-option” patients, with many studies demonstrating the potential of stem cells to enhance revascularization by promoting vessel formation and blood flow recovery in ischemic tissues. Herein, we provide an overview of studies focused on the use of angiogenic cell therapies in CLI in the last years, from approaches testing different cell types in animal/pre-clinical models of CLI, to the clinical trials currently under evaluation. Furthermore, recent alternatives related to stem cell therapies such as the use of secretomes, exosomes, or even microRNA, will be also described.

## 1. Critical Limb Ischemia

Critical Limb Ischemia (CLI) constitutes the most severe form of Peripheral Arterial Disease (PAD), a prevalent manifestation of atherosclerosis which involves the blockade of major systemic arteries other than those of the cerebral and coronary circulation [1], more common in legs than in arms [2]. PAD affects around 10–15% of adults, being an underestimated and underdiagnosed cardiovascular disease (CVD) due to its asymptomatic initial stages [3]. PAD is associated with risk factors such as older age, hypertension, dyslipidemia, or smoking [4], and it is more prevalent in diabetic people due to metabolic alterations such as angiogenesis impairment, inflammatory progression, or endothelial dysfunction [5,6,7,8]. CLI itself has an annual incidence of 0.35% and an average prevalence of 1.33%, affecting to 500–1000 people per 1 million population in Europe and the United States [9]. CLI patients are classified based on clinical criteria and hemodynamic parameters (i.e., pulse volume recordings, ankle and toe pressure values, rest pain, and tissue loss) [10,11,12] currently accepted in international consensus guidelines on PAD and CLI [12,13,14,15,16]. Overall, CLI patients suffer from chronic ischemic rest pain, ulcers, or gangrene, as well as an increased risk of cardiovascular events. CLI has a huge impact on the patients’ quality of life, being associated with an increased risk of amputations (fingers, toes, or extremities) and, moreover, an increase in mortality rates [15,17,18,19,20]. This debilitating disease causes high dependency on caregivers, requiring permanent local wound treatment, and the chronic use of pain-relieving medications, considerably diminishing patient’s quality of life [21].

Nowadays, the treatment of CLI remains highly variable and, in many situations, suboptimal [22]. Initial recommendations for CLI patients to prevent further cardiovascular events include smoking cessation, lipid lowering (statins mainly), antiplatelet therapies, or ACE inhibitors [16]. Alternatively, other medical strategies or pharmaceutical agents have been applied for the specific treatment of CLI patients (sympathectomy or spinal cord stimulation, iloprost) [23]. Unfortunately, these strategies do not seem to be totally effective in reducing limb-specific events [16], although larger studies/clinical trials are required in order to reach definitive conclusions.

The majority of CLI patients require revascularization interventions like bypass or angioplasty, having observed a significant improvement in the techniques and devices applied (cryoplasty, stent-grafts, drug-eluting balloons or stents, etc.) in the past decades. Nevertheless, the percentage of patients eligible for these strategies is not higher than 45% due to high comorbidity or surgical related issues such as difficult access due to narrow vessels, etc. Furthermore, patients that undergo surgery will usually require amputation at the short term [24]. Amputation rates are unacceptably high, typically exceeding 15–20% at 1 year and can vary by the presence of comorbid conditions [25] such as diabetes mellitus (DM), which elevates this rate up to 50% in CLI diabetic patients [26]. Diabetic patients have higher risk of suffering PAD/CLI and a negative outcome partly related to the abrogation of new vessel formation and remodeling of the pre-existing vasculature under hyperglycemic conditions [27]. Unfortunately, the increasing prevalence of PAD together with higher presence of other CLI risk factors (i.e., diabetes) and the rising number of people in advanced age provide little reason to believe that the number of patients suffering this disease will decrease in the near future [25]. The poor prognosis of CLI patients as well as their impaired quality of life makes compulsory to find effective and less invasive treatments. Moreover, the desirable treatment should be applicable to all CLI patients, because the actual percentage of ineligible patients is unacceptably high.

As an alternative to conventional treatments, therapeutic angiogenesis has arisen as a promising treatment for CLI patients, mainly those considered as “no-option”, due to the potential of this strategy to promote revascularization of ischemic tissues [28,29,30,31,32,33]. To date, different approaches including angiogenic gene or cell-based therapies are currently under investigation.

**Figure 1:**
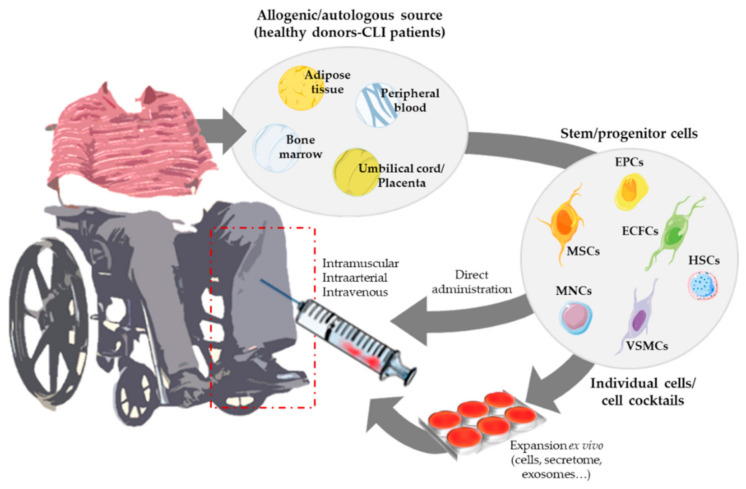
Overview of angiogenic cell therapy for Critical Limb Ischemia (CLI).

In this review, we have mainly focused on the use of angiogenic cell therapy for CLI (Figure 1), from animal/pre-clinical models designed to study CLI and the tools applied to test for revascularization in response to cell therapy, to the angiogenic therapies currently under evaluation in clinical trials. Moreover, recent alternatives derived from stem cell therapies, such as the use of secretomes, exosomes, or even microRNAs, will be described.

## 2. Animal Models of CLI

CLI animal models are not only used to study the disease itself [34,35], they also provide the appropriate scenario to evaluate strategies to induce neovascularization or to reduce inflammatory response. These models allow us to follow-up cell mobilization in response to ischemia [36,37,38,39]. Moreover, biodistribution assays are essential to determine the cell’s fate [40,41] and more importantly, to evaluate the biosafety profile, being required by regulatory guidelines prior to initiating cell therapy into the clinic [42]. Furthermore, for treatments testing human components such as human cells, immunosuppressed animals (nude, athymic, etc.) are usually applied [43].

**Figure 2:**
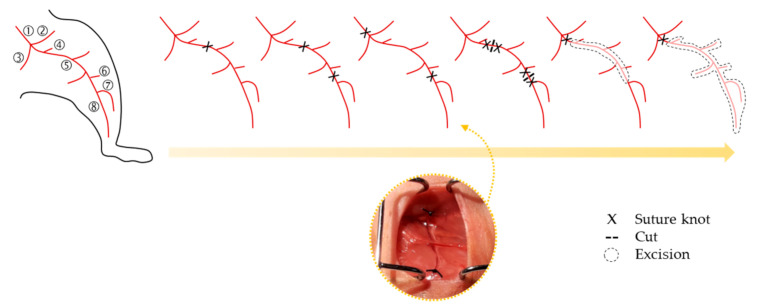
Schematic representation of femoral artery ligation (FAL) strategies usually applied to create PAD/CLI models, from the lowest (left) to the highest (right) severity models of the disease. A representative image of the FAL strategy followed in our research group is also shown [41,47]. Legend: ➀ Iliac artery, ➁ Iliacofemoral artery, ➂ Internal iliac artery, ➃ Pudendoepigastric trunk, ➄ Femoral artery and its branches (lateral circumflex and proximal caudal), ➅ Superficial caudal epigastric artery, ➆ Popliteal artery, and ➇ Saphenous artery. Arterial anatomy information was based on Kochi et al. [48].

Thus, in order to pre-clinically evaluate the effect of cell therapy on revascularization, it becomes essential first to be able to achieve an optimal model of CLI capable to resemble as much as possible the characteristics found in humans. Until now, femoral artery ligation (FAL) remains the most common approach to induce CLI, which is usually performed in one limb, leaving the other as a non-ischemic control. Several studies performing single or double femoral ligation, or alternatively cutting the femoral artery in different sites or even excision of the artery (partly or in all branches) can be found, creating different grades of CLI [44,45] (Figure 2). Additionally, depending on the occlusion site, extent of the injury or the occlusion tools (suture knots, constrictors, electrocoagulation, etc.), it is possible to create different degrees of the disease, causing different ischemic stages and patterns of perfusion restoration [43,46].

The resulting CLI model not only depends on the methods described to promote ischemia, but also on the operator performing the interventions, the animals used (mouse, rat, rabbit, pig, etc.), or even the strain selected [49,50,51]. Moreover, it is difficult to reproduce an animal model that resembles 100% CLI in humans, as this disease courses with a very slow progression, without important or aggressive symptoms for years, until becomes chronic. In this regard, Lejay et al. proposed a sequential ligation process, ligating first the femoral artery and days after the iliac artery, in order to achieve a progressive model and with similar impaired functions than patients [52]. Krishna et al. performed a “two-stage model”, with an initial arterial narrowing using ameroid constrictors over 14 days, prior to the induction of acute ischemia by FAL and excision [53]. Similarly, Han et al. created a model with local thrombosis in vessels by photochemical reaction triggered by the administration of erythrosine B, modifying endothelial function and occluding the vessels lumen by the blood clot, therefore getting closer to the human pathology than ligation [54].

On the other hand, the fact that most studies use healthy animals to generate CLI models constitutes an issue itself. CLI patients present, among other characteristics, endothelial dysfunction or reduced vascularity, which correlate with impaired vascular recovery. In FAL models, however, the vascular regeneration properties remain intact, which removes us from the reality of the patients’ symptoms. Moreover, autologous cell therapy appears to be less effective than expected because cells show impaired functions under pathological conditions. For that reason, researchers have tried to combine FAL with additional strategies to replicate the pathophysiological characteristics found in CLI patients. Parikh et al. combined FAL with endothelial nitric oxide synthase (eNOS) inhibitor administration, increasing vasoconstriction and ischemia by blocking nitric oxide (NO) production [55]. Alternatively, animal models presenting risk factors associated with PAD, such as hyperlipidemia, hypercholesterolemia, or diabetes, have also been employed. Thus, CLI models generated in hyperlipidemic and diabetic mice generally coursed with reduced collateral formation and blood flow recovery, showing better correlation with human patients [56]. Apolipoprotein E (ApoE)-deficient mice, commonly accepted as a model of atherosclerosis [4], also show a decrease of muscle regeneration after FAL surgery [37].

### Strategies Followed to Assess Neovascularization in CLI

**Figure 3:**
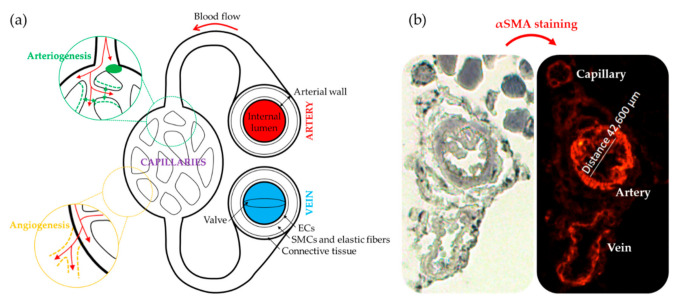
Mechanisms of neovascularization. (**a**) Schematic representation of the circulatory system in which angiogenesis and arteriogenesis processes are represented. (**b**) Representative immunohistochemistry image of blood vessels detected in the low back muscle of a CLI mouse [47], to evaluate vascular density and diameter size using anti-smooth muscle α-actin (α-SMA, red) antibody.

The ultimate goal of any CLI treatment is to promote post-natal neovascularization, a repairing mechanism that takes place in response to ischemic events as an strategy to recover the damaged tissues and provide sufficient oxygen and nutrient supply to ensure tissue surveillance [57]. In adults, neovascularization comprises both angiogenesis and arteriogenesis, processes in which different types of vascular and immune cells participate [58]. Angiogenesis consists in the formation of new blood vessels from existing ones, while arteriogenesis involves collateral growth and remodeling of pre-existing arterioles to generate larger conductance vessels and to compensate for the loss of blood flow of occluded arteries (Figure 3a) [59]. Remarkably, FAL animal models and patients usually show similar neovascularization patterns, with enhanced arteriogenesis next to the occlusion site and increased angiogenesis in the distal ischemic tissue [49]. Thus, therapeutic strategies should seek the stimulation of both processes in order to promote neovascularization [58].

In studies involving CLI animal models (Table 1), different strategies are usually applied to analyze potential neovascularization. Blood flow recovery over time is often analyzed by Laser Doppler Perfusion (LDP). This technology is based on Doppler effect, consisting in the alteration of a wave’s frequency as result of the movement between a laser light and circulating red blood cells. Alternatively, the LDP Imaging system creates images from blood perfusion values per pixel, getting a map of the blood flow in the region of interest [49]. In FAL-based studies, perfusion is measured before and after surgical intervention, and then registered during several days, usually 3–4 weeks. Perfusion data are normally shown as blood flow ratios (ischemic limb/healthy limb). Although there are other techniques to evaluate collateral formation and limb perfusion such as X-ray micro-angiography [49], LDP has been the most applied tool in recent publications due to the easy handling of the equipment and, moreover, because it constitutes a noninvasive method.

Histological analysis by immunohistochemistry (IHC) is also used to evaluate angiogenesis and arteriogenesis post-mortem. Most studies use anti-α smooth muscle actin antibodies to identify blood vessels in tissues (Figure 3b), together with antibodies against endothelial cells markers, such as CD31, von Willebrand factor, or lectins with specific affinity for endothelial cells, like *Ulex europaeus* agglutinin I in humans or *Griffonia simplicifolia* lectin I isolectin B4 in non-primates [47,49,60,61]. For angiogenesis, vascular density is calculated by counting the number of blood vessels, and capillary diameters are measured for arteriogenesis evaluation. The internal lumen’s diameter is normally measured to evaluate arteriogenesis, although the arterial wall area is also interesting since arteriogenesis increases diameter and wall thickness [59,62]. Results are usually expressed as the number of blood vessels per mm^2^ in angiogenesis and blood vessel diameter (µm) or area (μm^2^) in arteriogenesis. Alternatively, another method to study angiogenesis is an in vivo matrigel plug assay, consisting in the injection of matrigel or similar hidrogels containing specific cell types into the subcutaneous space [63]. After several days of post-implantation, mice are sacrificed and the matrigel plugs are extracted and excised for further analysis. Sections can be then stained to identify capillary structures, and vasculature growth into matrigel provides information regarding angiogenesis [49].

## 3. Angiogenic Cell Therapy

Angiogenic therapy involves the use of angiogenic growth factors (VEGF, HIF-1a, FGF1, HGF, etc.) [33,64], gene transfer techniques using viral or non-viral vectors to transport a gene codifying for a therapeutic protein to the target tissues [65] or, alternatively, the use of angiogenic stem cells. All these strategies aim to improve revascularization by increasing the number/size of blood vessels, promoting blood flow recovery and therefore increasing tissue perfusion in the ischemic extremities [65]. Among them, cell-based therapies seem more efficient compared to protein- or gene-based approaches, not only because of their direct vasculogenic properties, but also due to their paracrine effect. Angiogenic cells can directly participate in the formation of new vessels, while in parallel they also provide endogenous growth factors, promoting vascular growth by paracrine fashion [66,67].

Thus, neovascularization can also be promoted by vasculogenesis, the novo formation of vessels mediated by circulating progenitors or stem cells [59]. Vasculogenesis was initially considered as an embryogenic process. However, post-natal vasculogenesis can also take place by incorporation of vascular stem or progenitor cells into vessel structures, allowing the formation of adult blood vessels [68]. To date, several strategies based on the use of stem and progenitor cells are being tested (Table 1), to promote vasculogenesis but also angiogenesis and arteriogenesis. The safety and efficacy of cell implantation therapies make of this less invasive treatment a feasible option for CLI patients.

### 3.1. Cell Therapies Based on Single or Combined Isolated Cells

Mesenchymal stem cells (MSCs) are the most used cells in advanced therapies for CVDs [96]. MSCs can be isolated from bone marrow, peripheral blood, or adipose tissues, and from them we can obtain osteoblasts, chondrocytes, adipocytes, neurons, endothelial cells (ECs), skeletal muscle cells, and vascular smooth muscle cells (VSMCs) [97]. MSCs are reported to promote angiogenesis because of their capacity to induce ECs proliferation, migration, and tube formation, while decreasing apoptosis and fibrosis [96,98,99]. Furthermore, MSCs support neoangiogenesis, releasing soluble factors that contribute to stimulate angiogenesis [100]. These cells are thought to improve hind limb ischemia by secreting cytokines that regulate macrophage differentiation to M2, an anti-inflammatory phenotype [101]. Likewise, apart from MSCs, endothelial progenitor cells (EPCs) also represent an important group of cells used in vascular regeneration. In 1997, Asahara et al. demonstrated that CD34+ cells can be isolated from peripheral blood mononuclear cells (PB-MNCs) and differentiated in vitro into ECs, showing the potential use for collateral vessel growth augmentation in ischemic tissues [102]. Although CD34 is not a specific marker of a single cell type, it is mostly associated to EPCs. Many researchers have explored the potential of using EPCs in tissue engineering as an angiogenic source for vascular repairing [103,104]. In the past years, several isolation and culturing techniques for EPCs have been described. Besides, the controversy regarding the definition of EPC phenotypes remains, with different studies still presenting a variety of results in terms of surface-based EPC markers [47,103,105,106]. At least, two different sub-populations have been accepted and clearly defined, based on their differentiation status and the capability to form colonies: early EPCs (eEPCs) also named circulating angiogenic cells (CACs) or myeloid angiogenic cells (MACs), with hematopoietic phenotype, and late EPCs or endothelial colony forming cells (ECFCs), with endothelial phenotype [106]. EPCs have been thought to derive from hematopoietic stem cells (HSCs), some EPCs could be derived from a niche close to the vasa vasorum in the macro-vascular wall [107]. Despite the controversy regarding the nature of these cells, no one denies the potential of EPCs to promote therapeutic angiogenesis and neovascularization of ischemic tissues [73,74,107]. Overall, in response to injury, cytokines and growth factors mobilize EPCs from the bone marrow into the peripheral blood, which will then participate in neovascularization [73]. Very recently, we have shown how, first days after administration of CACs to ischemic CLI mice, these cells migrate into the ischemic tissues, modulating immune cells recruitment and promoting an increase of angiogenesis and arteriogenesis [47]. However, the administered cells do not remain in the ischemic tissues over time suggesting that they may promote vasculogenesis in a paracrine form [47,108]. Moreover, early EPCs do not seem to differentiate to ECs, with this role being assigned to ECFCs [106,109]. Indeed, different studies support that the regenerative properties of eEPCs are mainly due to paracrine effects, while ECFCs present vessel-forming activity in vivo [47,109]. Thus, a cell therapy mediated by both cell types, early, and late EPCs, could be a good strategy for CVDs. Yoon et al. evaluated this combined cell therapy, demonstrating a synergistic neovascularization involving several cytokines and matrix metalloproteinases (MMPs) [77]. Very recently, our group has also corroborated the potential of CACs to promote angiogenesis of ECFCs in vitro, and such effect was impaired under an atherosclerotic environment [110]. In the same way, different cell combinations have been tested. Rossi et al. demonstrated that co-injection of MSCs with ECFCs in a murine model of CLI increased vessel density and foot perfusion in greater ratio than cells individually administrated; corroborating the theory that MSCs support ECFC-mediated angiogenic processes [72]. Furthermore, their results indicated that MSCs accelerated muscle recovery via endoglin dependent mechanism. Similarly, the combination of EPCs and smooth muscle progenitor cells (SMPCs) has also been evaluated to treat CLI. This cell mixture improved vascular network formation, with both ECs and smooth muscle cells (SMCs) participating in vessel maturation and stability. Likewise, Foubert et al. demonstrated that co-administration of EPCs and SMPCs activates neovascularization resulting in a more effective therapy than these cells administrated separately [78]. Some studies suggest that SMCs may also originate from bone marrow-derived cells as SMPCs have been identified in peripheral blood [111].

### 3.2. Cell Therapies Based on Cellular Cocktails

As an alternative to the injection of a single cell type or the combination of two previously isolated cells, the administration of cellular cocktails derived from different niches, such as bone marrow, peripheral blood, or adipose tissue, is also a frequent approach to treat CLI. Indeed, the regenerative properties of mononuclear cells (MNCs) derived from either bone marrow or peripheral blood have been largely studied in the last years. Therapies employing bone marrow mononuclear cells (BM-MNCs) constitute a promising alternative for CLI patients to avoid or delay the onset of amputation [112]. BM-MNCs consist of a heterogeneous mix of multipotent stem cells working cooperatively as MSCs, HSCs, EPCs, monocytes, lymphocytes, and pluripotent stem cells [41,113]. We and other researchers have reported the beneficial effects of different combinations of BM-MNCs, representing an effective approach in promoting new vessel formation, perfusion recovery, and CLI reversal [41,100,114,115,116,117,118,119,120,121,122]. In the ischemic tissue, BM-MNCs produce and secrete different cytokines and growth factors [123] and increase neovascularization and collateral vessel formation in limb ischemia [79]. Moreover, Kikuchi-Taura et al. have recently described that transplantation of BM-MNCs into a murine stroke model promoted ECs angiogenesis by gap junction mediated cell–cell interactions, elucidating a new theory of how cell-based therapies work, and suggesting that stem cells supply energy to injured cells [124]. This study suggested that, under hypoxic conditions, transplanted BM-MNCs are capable to transfer small molecules to ECs via gap junction interactions, leading to HIF-1α activation, which induced upregulation of VEGF uptake into ECs and ECs autophagy suppression [124].

Alternatively to BM-MNCs, PB-MNCs are formed by circulating cells with angiogenic potential, thereby several studies involving the administration of these cells to treat CLI have also shown promising results [125,126]. Li et al. made a comparison between CD34+ and CD34- cells in PB-MNCs, concluding that both induce neovascularization, but only CD34+ incorporate into new capillaries [89]. PB-MNCs promote revascularization in ischemic limbs, even more when they are combined with platelet-rich plasma (PRP) [90]. PRP, a source of platelets, cytokines, and growth factors, participates in ECs proliferation and differentiation, interacting with important cell receptors related with angiogenesis [90]. Furthermore, in order to achieve high stem cell concentrations, hematopoietic growth factors are frequently used to induce cell mobilization. For example, prior PB-MNCs harvesting, progenitor cells are usually mobilized injecting granulocyte colony-stimulating factor (G-CSF) [125,126,127,128]. BM-MNCs and PB-MNCs treatments have been compared, and no significant differences have been observed between them [129,130]. Remarkably, without previous mobilization, PB-MNCs show higher concentration of mature cells as red blood cells, platelets, lymphocytes, and monocytes, while BM-MNCs show higher levels of EPCs [131].

The use of adipose tissue-derived stem cells (ASCs) has increased in the last years, due to the easier accessibility, abundance, and less painful collection compared to other sources such as bone marrow [132]. The stromal vascular fraction (SVF) derived from adipose tissue contains heterogeneous cell populations such as mesenchymal progenitor/stem cells, pre-adipocytes, endothelial cells, pericytes, T cells, and M2 macrophages. SVF-derived mesenchymal progenitor/stem cells, usually referred as ASCs themselves, can be easily expanded in vitro and have the potential to differentiate into multiple lineages, including myogenic, osteogenic, neurogenic, and hematopoietic pathways [133,134,135,136,137]. The angiogenic properties of these cells have been correlated with a strong paracrine activity, secreting an important number of angiogenesis-related cytokines [136]. Moreover, the administration of ASCs to CLI mice promotes a significant recovery of blood flow in ASCs treated mice compared to ischemic, non-treated ones [133]. Very recently, Liu J et al. have shown that the regenerative properties of transplanted ASCs might correlate with an immunomodulatory effect promoted by these cells. In presence of ASCs, a higher number of macrophages can be found in the muscle, with increased presence of M2 macrophages [91], and its administration in a murine model of CLI induces an angiogenic process in the ischemic tissue [133]. The clear advantages of using these cells are easy access and isolation. ASCs are highly abundant in adipose tissue, making almost unnecessary culture expansion of these cells. Moreover, adipose tissue harvesting requires a minimally invasive intervention [138]. A pilot study using adipose-derived regenerative cells (ADRCs) in CLI patients has been recently published [139].

Finally, other cells with multi-differentiation potential such as amniotic fluid derived stem cells (AFSCs) or umbilical cord blood and placenta tissue derived stem/progenitor cells have also been considered. Placenta-derived MSCs stromal-like cells (PLX-PAD) in CLI mice are currently being tested in a Phase III trial (PACE Trial) with atherosclerotic CLI patients (NCT03006770) after promising results in animal assays [95]. Unfortunately, the low availability of these cells together with ethics concerns related to their use, has limited their translation as cell therapies.

## 4. Clinical Trials

The exciting results derived from pre-clinical studies fomented the initiation of numerous clinical trials: to date, over 50 studies have investigated a variety of cell therapies, usually employing BM- or PB-derived MNCs, showing modest but significant improvements of ischemic symptoms [140,141]. Patients enrolling these trials normally suffered from severe stages of PAD (Fontaine III-IV) with pain at rest due to atherosclerosis obliterans (ASO) rather than thrombo-angiitis obliterans (TAO) or Buerger’s Disease. The first clinical trial that reported the efficacy of autologous BM-MNCs administration as cell therapy for CLI was published in 2002 [142]. The Therapeutic Angiogenesis using Cell Transplantation (TACT) trial conducted a pilot study first with 25 patients, followed by a randomized controlled trial in which 22 patients with bilateral leg ischemia were injected with BM-MNCs in one leg and PB-MNCs in the other as controls. Their findings indicated a significant improvement in ankle-brachial index (ABI), transcutaneous oxygen pressure (TcPO_2_), and pain-free walking time sustained at 24 weeks, with a limb status improved in 39 out of 45 patients [142]. Table 2 includes a list, far from complete, of clinical studies already completed and with results published in the past decades, involving the use of different cell types, cell doses, and administration routes, with a minimum of 10 patients enrolled. Due to the huge interest in the field, the number of ongoing clinical trials using cell therapy in CLI is constantly growing, including examples such as the Phase III PACE trial (PLX-PAD cells, NCT03006770) [143], or the Phase III trial testing Rexmyelocel-T (REX-001), a solution enriched with human BM-derived MNCs (NCT03174522 and NCT03111238) in CLI Rutherford V and DM patients. Some of these studies are active and recruiting, therefore, their results are not yet available. Additional information regarding such trials can be found at www.clinicaltrials.gov (accessed on 14 December 2020).

One of the primary outcomes seen in clinical trials is hemodynamic improvement, represented as an absolute increase of ABI >10% [12,164]. Similarly, other researchers have reported an enhanced blood perfusion when administering either BM-MNCs, PB-MNCs, or MSCs [114,125,127,128,145,147,150,153]. Gupta et al. evaluated the effect of allogenic MSCs in patients (Rutherford grade 4–6) that suffered from CLI due to ASO and TAO. This study reported a significant increase of ABI (*p* = 0.0018) after 6 months of MSCs treatment (n:10) compared to patients transplanted with placebo (n:10), although no such significant changes could be observed in rest pain, ulcer healing, or amputation rates. Some authors debate about the selection of ABI as a primary endpoint, as this parameter is not considered a useful predictor for evaluating the long-term efficiency of the angiogenic therapy using bone marrow cells [20,142]. The PROVASA study, a randomized, double-blind, placebo-controlled intra-arterial progenitor cell transplantation of BM-MNCs for induction of neovascularization in patients with PAD, showed no significant differences in ABI primary outcome at 3 months. However, authors did observe significant improvements in other secondary endpoints, like ulcer healing and rest pain reduction in the BM-MSCs group [20].

Luckily, cell therapy has promoted an amelioration of the symptoms and therefore an improvement in the quality-of-life of these patients [158,160]. Thus, an improvement in rest pain is defined as a >50% decrease in pain scores, assessed with the visual analogue scale (VAS) at different time points [165]. In a non-randomized study, CLI-TAO patients (n:40) received autologous BM-MNCs, and after a mean follow-up of 129 months, a prominent improvement in VAS (*p* = 0.0001) was seen, also in their primary endpoint amputation-free survival and other secondary outcomes including ulcer status, ABI, toe-brachial index, and TcPO_2_ [116]. This last hemodynamic measurement is employed in several clinical trials as secondary endpoint, and an augmentation in oxygen pressure has been observed in numerous studies using PB-MNCs, BM-MNCs, MSCs, as well as peripheral blood-derived angiogenic cell precursors (Ves-cells) [20,114,116,128,129,142,145,150,153].

Alternatively, CD34+ or CD133+ isolated cells have also been tested in CLI studies. In a randomized single-blinded non-inferiority trial, patients were divided 1:1 into those receiving either PB-MNCs or purified CD34+ cells. Although the number of patients included in this study was low, similar results were found in terms of limb salvage and quality of life improvements. No significant differences were found between both treatments in terms of amputation-free survival. On the other hand, the CD34+ group seemed to achieve faster rest-pain relief and overall earlier ischemia relief than the PB-MNCs group [166]. In the Stem Cell Revascularization for Patients with Critical Limb Ischemia (SCRIPT-CLI) trial, subjects with CLI due to ASO were divided in two groups in 2:1 proportion, receiving an active treatment with G-CSF for 5 days before leukapheresis and CD133+ injection in both legs, and a group receiving saline injections plus a sham leukapheresis and a placebo-buffered solution instead of cells. The safety of the procedure was proven 12 months after treatment, whereas a poor mobilization of CD133+ cells was found in several patients, together with higher rates of CD133+ senescent cells. These results reflected the need of studies with higher number of patients. Nevertheless, these authors suggested that this therapeutic approach might not be entirely successful with the patients selected [154]. Finally, other type of cells gaining popularity as a potential treatment for CLI patients are ASCs, SVF, or the very small embryonic-like stem cells (VSEL) [139,167,168].

### Limitations in Cell-based Clinical Trials

Despite the promising results derived from the use of stem cells with CLI patients, the variability and heterogeneity found within the clinical trials is high. Remarkably, after 20 years of using stem cell therapy in CLI, it remains unclear which cell type or cell source triggers the highest benefits in terms of blood perfusion recovery or amelioration of ischemic symptoms. The first trials focused on using heterogeneous cell preparations from either bone marrow of peripheral blood. Such unpurified cell mixtures are often composed by a considerably low proportion of “active” cells, or cells with documented pro-angiogenic functions [109,125,142,169]. On the other hand, large amounts of cells are required during cell therapy, but proangiogenic progenitor cells are not present in high proportions in humans, being necessary to develop optimized and clinically applicable culture expansion methods for future perspectives. Cells could be efficiently selected by their expression of CD34, CD133, or also by their ALDH-activity, although this approach have detractors too, as extended culture is thought to negatively affect cell regenerative function [170,171]. However, the optimization of these cultures could solve the problematic associated with EPCs or MSCs dysfunctionality in CLI patients, as well as augmenting the angiogenic potency of cells through pre-stimulation prior transplantation [47,172]. In this sense, the next question relies in whether using an autologous or an allogenic strategy with these patients. Autologous administration avoids rejection-related issues, but also presents several disadvantages such as difficulties to recruit a significant number of cells from these donors, and moreover the already mentioned cellular dysfunction in response to atherosclerosis and/or related co-morbidities. The allogenic method complicates the therapy applicability by requiring HLA-matching [173,174,175].

Another matter of disparity is the cell dose to apply, depending on the cell type/source, as the number of MNCs and purified cells from MNCs always vary between patients and could be affected by the illness itself. Unfortunately, some studies did not even provide such information [30,170,174]. Similarly, the route of administration has not reached a consensus yet. The majority of trials have chosen an intramuscular cell delivery, considering this a more feasible and less invasive strategy [147,150]. Other authors postulate that an intra-arterial administration would better distribute the cells into areas with sufficient oxygen to prolong the pro-angiogenic function, trying to avoid the transient cell engraftment and integration after intramuscular injection [20,87,148]. In this regard, several studies have compared both intramuscular and intra-arterial strategies, showing similar results in terms of clinical outcome [176,177,178].

Overall, the comparison of the results derived from different trials comprises an arduous task in which meta-analysis are becoming increasingly useful to support evidence-based medicine, allowing to summarize the accumulated evidence and also to drive future research [179]. A meta-analysis performed by Rigato et al. includes robust statistical analysis of either randomized, controlled trials, and non-controlled studies. Their results showed that in patients not eligible for surgical revascularization, autologous cell therapy has the potential to reduce the risk of major amputation in 36%, improving also the probability of wound healing in 59%. Moreover, it appears to ameliorate several surrogate endpoints of limb perfusion, pain, and functional capacity [180]. Similarly, Gao et al. analyzed the results of over a thousand patients enrolling randomized controlled trials, indicating that cell implantation improved ulcer healing rate, ABI, TcPO_2_, pain-free walking distance, and reduced amputation rate and rest pain score compared with standard care/conventional treatment [181]. Very recently, a review including 11 meta-analyses evaluated current evidence on cell-based therapy in PAD. Such study corroborates the effectiveness of using cell therapy with CLI patients, with a reduction in the number of major amputations and improved wound healing. Furthermore, for secondary outcomes such as ABI, TcPO_2_, and RPS, a general improvement is seen [179]. Despite this, larger studies are required to increase statistical significance, together with the design of placebo-controlled studies, as clinical outcome differences are not clear when compared to the placebo effect [181,182].

The discrepancy found between the clinical trials reflects the fact that there is still a lot of work to do, as stated before, in order to reach a consensus regarding the optimal treatment. This, in turn, also requires of a better understanding of how cells work, in order to implement their use in clinical practice. Still, these studies share a common conclusion: the safety and feasibility of cell therapy in patients with no option of surgical revascularization, a population that represents half of the CLI patients diagnosed [120,144].

## 5. Strategies Derived from Cell Therapy

Due to increasing number of studies supporting that the regenerative power of stem cells is mainly due to their paracrine effect within the ischemic tissues, the use of the cells released factors (secretome) and, more recently, the so-called exosomes as an alternative to cell therapy, is currently being investigated. Secretomes are also named in different studies as conditioned medium (CM), referring to the factors released to the medium where cells have been cultured. The modulatory effect of these secretomes could depend on the presence of different growth factors, angiogenic factors, hormones, cytokines, extracellular matrix proteins and proteases, hormones, lipid mediators and genetic material secreted from stem or progenitor cells for cell communications, interfering in different biological functions such as growth, division, differentiation, apoptosis, and signaling [183,184]. The stem cell secretome has shown great potential and could mediate intracellular pathways in injured cells or activate adjacent tissues secretion [184].

Secretomes derived from different progenitor or stem cells are being studied, especially thanks to mass spectrometry approaches. In this way, Barberg et al. analyzed the MSCs secretome composition, identifying proteins related to cell growth, signal transduction and cell communication, as well as cytokines and growth factors involved in physiological regulation of hematopoiesis [185]. Likewise, Maffioli et al. described that, in a proinflammatory environment, MSCs increase the secretion of proteins related with immunomodulation and angiogenesis [186]. Although MSCs secretomes are the most studied ones, secretomes of other stem/progenitor cells are also showing promising results. Very recently, we analyzed by a proteomic approach the secretome of CACs, identifying a significant number of angiogenic factors, and moreover, we demonstrated that incubation ex vivo of ECFCs with this secretome enhances ECFCs angiogenesis, in agreement with previous studies [77,110]. Moreover, ASCs secretome contains multiple angiogenic factors, which appear to promote, among others, survival, proliferation, and migration of ECs, as well as vasculogenesis [187,188]. Indeed, ASCs conditioned medium has been shown to enhance proliferation and survival of endothelial cells in vitro [133]. Some of these secretomes have already been tested as therapy in vivo showing encouraging results, since they seem to be as effective as cell therapy [189,190,191]. The complete knowledge of the secretomes activity and their factors would allow us to reproduce them artificially by means of bioactive molecules to use in regenerative medicine. Finally, the administration of secretomes as an alternative approach to cell therapy eliminates disadvantages such as immune rejection or tumorigenicity [184]. Currently, novel strategies such as secretomes liberation approaches to enhance their angiogenic properties are being evaluated. For example, Felice et al. used nanoparticles to achieve a controlled EPCs secretome, demonstrating the potential of this system in FAL rat models [192]. Likewise, extracellular vesicles derived from stem or progenitor cells, also called “exosomes”, seem to participate as well in the regenerative role of cellular secretomes. Exosomes derived from MSCs appear to promote bone regeneration and angiogenesis [193]. In the same way, exosomes derived from CD34+ cells have been shown to participate in angiogenesis and are essential for the repairing properties assigned to these cells [194].

Finally, microRNAs have recently arisen as a promising alternative therapy against ischemic diseases. MicroRNAs, short non-coding RNAs that inhibit translation of messenger RNAs, can regulate an entire network or pathway simultaneously, besides, in response to ischemia, they appear to be involved in the regulation of angiogenesis and arteriogenesis [58,195]. Different strategies against PAD are based on the modulation of factors related to the development of vasculature. However, modifications in a single factor do not seem to be sufficient for the treatment of this disease, and therefore the development of therapeutic strategies based on microRNAs are very promising, as this approach would allow to regulate several pathways at the same time. Some of the most studied microRNAs in CLI are miR-494, miR-487b, miR-329, and miR-495. Thus, the inhibition of some of these molecules, described as antiangiogenic microRNAs, seems to promote blood flow recovery in CLI mice [195,196]. Some studies suggest that microRNAs could be transferred by stem or progenitor cells through exosomes to ECs, promoting angiogenesis in these forms [197]. Although microRNAs are the best known and most studied RNA non-coding molecules for their therapeutic potential, there are other related types of RNA, such as circular or long noncoding RNAs, that also act in the regulation of gene expression and therefore should be also evaluated as therapeutic targets.

## 6. Conclusions

In the past decades, an enormous effort has been made to find appropriate strategies for the optimal treatment of CLI patients. Stem cell-based therapies have proven to be safe and efficient to achieve therapeutic angiogenesis and to promote blood flow recovery, representing an alternative for these patients. In this sense, the interest in the field is clear, and the number of clinical trials using cell therapy in CLI is constantly growing. Still, the variability seen between these trials is high, reflecting a lack of consensus regarding key factors such as cell doses, cell types or sources, administration routes, the parameters to define outcome efficacy, or the cohorts themselves. Moreover, further investigation is required in order to better understand how the cells, or the molecules/exosomes derived from them, exert such beneficial effects. Thus, a lot of work needs to be done before their translation into the clinical practice. Even so, the results are promising, and a therapy based on the administration of stem/progenitor cells and/or their derivatives could hopefully represent a good alternative for CLI patients, especially for those with no other options.

## Figures and Tables

**Table 1:** Classification of most important cell therapy pre-clinical studies. The table includes cell type used, first author and year of publication, reference number (ref), cell source, animal and strain, number of administered cells, route of administration, follow-up, and parameters checked to evaluate the therapy outcome. Abbreviations included (alphabetical order): a: autologous; AD: Arteriolar density; AI: Angiographic index; BFP: Blood flow perfusion; CBP: Calf Blood Pressure; CD: Capillary density; CVF: Collateral Vessel Formation; ESC-ECP: Stem cell-derived endothelial cell product; FS: Functional score; h: human; IA: Intraarterial; IC: Intracardiac; IM: Intramuscular; IV: Intravenous; MP: Matrigel plug; SC: Subcutaneous; TR: Tissue regeneration; VD: Vessel diameter; VIP: Vascular intersection percentage; VS: Visual Scale

Cell Type	Author (Year)	Ref.	Cell Source	Animal (Strain)	Administration (×10^5^ Cells)	Route of Administration	Follow-up (Weeks)	Outcome
**aMSCs**	Cunha (2013)	[69]	Bone marrow	Mice (Balb-C & C57/BL6)	5	IM	4	VS, CD, TR
**hMSCs**	García-Vazquez (2019)	[70]	Adipose tissue	Mice (Athymic nude)	6	IM	3	BFP, CD, VS
**aMSCs**	Nammian (2021)	[71]	Bone marrow & adipose tissue	Mice (C57/BL6)	5	IM	4	FS, CD
**hMSCs + hECFCs**	Rossi (2017)	[72]	Bone marrow & peripheral blood	Mice (Athymic nude)	N/A	IV	2	BFP, CD, VS
**hCD34+**	Lian (2018)	[73]	Peripheral blood	Mice (Balb-C Nude)	1	IM	3	FS, VS
**hEPCs**	Kalka (2000)	[74]	Peripheral blood	Mice (Athymic nude)	5	IC	4	BFP, CD, VS
**hEPCs**	Urbich (2003)	[75]	Peripheral blood	Mice (Athymic NMRI Nude)	5	IV	2	BFP, CD
**hEPCs**	Zhao (2016)	[76]	Fetal aorta	Rat (Goto-Kakizaki)	100	IM	8	BFP, CD, VS
**hCACs**	Beltrán-Camacho (2020)	[47]	Peripheral blood	Mice (Balb-C Nude)	5	IM	4 days	BFP, CD, FS, VD
**hEPCs + hOECs**	Yoon (2005)	[77]	Peripheral blood	Mice (Athymic nude)	2	IM	3	BFP, CD, VS, MP
**hEPCs + hSMPCs**	Foubert (2008)	[78]	Umbilical cord blood	Mice (Athymic nude)	5	IV	2	BFP, CD, AD
**hESC-ECP**	MacAskill (2018)	[40]	hESC line	Mice (CD1-STZ DM inductor)	10	IM	3	BFP, CD
**aBM-MNCs**	Shintani (2001)	[79]	Bone marrow	Rabbit (Male New Zealand White)	5	IM	4	BFP, CBP, CD, CVF
**aBMCs**	De Nigris (2007)	[80]	Bone marrow	Mice (ApoE−/−)	20	IV	2	BFP, CD, CVF
**aBM-MNCs**	Jeon (2007)	[81]	Bone marrow	Mice (C57/BL6)	20	IM	4	CD, CVF
**aBM-MNCs**	Gan (2009)	[82]	Bone marrow	Mice (C57/BL6)	30	IM	2	BFP, CD
**hBM-NCs**	Liu (2009)	[83]	Bone marrow	Mice (C57/BL6 ApoE−/−)	250	IA	4	BFP, CVF
**aBM-MNCs**	Brenes (2012)	[84]	Bone marrow	Mice (C57/BL6)	5, 10 & 20	IM	4	BFP, CD, FS
**aBM-MNCs**	Reis (2014)	[85]	Bone marrow	Mice (Balb-C)	5	IM	4	CD, TR, VS
**hBM-MNCs**	Rojas-Torres (2020)	[41]	Bone marrow	Mice (Balb-C Nude)	10	IM	3	BFP, CD, FS, VD
**aBMC-derived macrophages**	Kuwahara (2014)	[86]	Bone marrow	Mice (C57/BL6N)	1	IM	4	BFP, CD
**hALDH high activity cells**	Capoccia (2009)	[87]	Bone marrow	Mice (NOD/SCID b2M)	1–2	IM	3	BFP, CD
**aMIAMI cells**	Rahnemai-Azar (2011)	[88]	Bone marrow	Mice (Balb-C)	10	IM	4	BFP, CD, FS, VS
**hPB-MNCs^1^**	Li (2006)	[89]	Peripheral blood	Mice (Athymic nude)	10	IM	4	BFP, AI, CD, VS
**aPB-MNCs + PRP**	Padilla (2020)	[90]	Peripheral blood	Rat (Wistar)	15	IM	4	AI, VIP
**aASCs**	Liu (2020)	[91]	Adipose tissue	Mice (C57/BL6)	10	IM	3	BFP, CD, VS
**aASCs + macrophages**	Rybalko (2017)	[92]	Adipose tissue	Mice (C57/BL6)	2	IM	3	BFP, CD
**hSVF**	Jin (2017)	[93]	Adipose tissue	Mice (Nude)	10	IM	2	BFP, VS, CD, MP
**PDX-PAD** **(adherent stromal cells)**	Prather (2009)	[94]	Placenta	Mice (Balb-C)	10	IM	3	BFP, CD, FS
**PLX-PAD** **(MSC like stromal cells)**	Zahavi-Goldstein (2017)	[95]	Placenta	Mice (C57/BL6)	0.02–10	IM & SC	3	BFP, VS

^1^ Cells mobilized with G-CSF.

**Table 2:** Classification of most important cell therapy clinical trials. The table shows first author and year of publication, reference number (ref), type of cell therapy, type of study, associated cause of PAD/CLI, disease stage, number of patients (T=Treated/C=Controls), control used, number of administered cells, route of administration, follow-up, and parameters checked to evaluate the cell therapy outcome. Parameters registering general improvement vs. baselines/controls are highlighted (**bold**). Abbreviations included (alphabetical order): ABI: Ankle-brachial index; AFS: Amputation-free survival; AR: Amputation rate; ASO: Arteriosclerosis obliterans; DR: Death rate; ECEPCs: enriched circulating endothelial progenitor cells; HD: High dose; LD: Low dose; MD: Medium dose; NC: Non-controlled; NR: Non-randomized; PFWD: Pain-free walking distance; RCT: Randomized controlled trial; RPS: Rest pain score; TAO: thromboangiitis obliterans; TcPO_2_: Transcutaneous oxygen pressure; UH: Ulcer healing.

Author (year)	Ref.	Type of Cell Therapy	Type of Study	Cause of PAD/CLI	Disease Stage	Nº Patients (T/C)	Control	Administration (x10^6^ cells)	Route of Administration	Follow-up (Months)	Outcome
**Huang** **(2005)**	[125]	PB-MNCs^1^	RCT	ASO	Fontaine III–IV	28 (14/14)	Blank	3000	IM	3	**ABI**, **AR**, DR, **PFWD**, RPS, **UH**
**Ozturk** **(2012)**	[128]	PB-MNCs^1^	RCT	N/A	Fontaine III–IV	40 (20/20)	Blank	24.8/mL (CD34+)	IM	3	**ABI**, AR, **PFWD**, **RPS**, **TcPO_2_**, **UH**
**Mohammadzadeh** **(2013)**	[127]	PB-MNCs^1^	RCT	N/A	Fontaine III–IV	21 (7/14)	Blank	900–1200	IM	3	**ABI**, **AR**, **UH**, **PFWD**
**De Angelis (2015)**	[144]	PB-MNCs	NR	ASO	Fontaine IV	86 (43/43)	Blank^5^	125.65	IM	4.5	AFS, **AR**, DR, PFWD, **RPS**, **UH**
**Tateishi-Yuyama** **(2002) TACT**	[142]	BM-MNCs	NR	ASO	Fontaine III–IV	25^2^	Blank	700–2700	IM	6	**ABI**, **TcPO_2_**, **RPS**
		BM-MNCs	R		Fontaine III–IV	22^3^	Placebo	889–2800	IM	6	
**Arai** **(2006)**	[145]	BM-MNCs	RCT	N/A	Fontaine III–IV	26 (13/13)	Blank	1000–3000	IM	1	**ABI**, **TcPO_2_**, RPS
**Dubsky** **(2013)**	[129]	PB-MNCs	NR	N/A	Rutherford 4–6	33 (11/22)^4^	Blank	10400	IM	6	**AR**, **TcPO_2_**, **UH**
		BM-MNCs				39 (17/22)^4^		1800			
**Huang** **(2007)**	[146]	PB-MNCs	NC	ASO	N/A	76	N/A	7200	IM	3	**ABI**, AR, PFWD, **RPS**, TcPO2, UH
		BM-MNCs				74		580			
**Matoba** **(2008)**	[30]	BM-MNCs	NC	ASO & TAO	Fontaine III–IV	115	N/A	N/A	IM	25.3	ABI, AFS, DR, **PFWD**, **RPS**, TcPO2, **UH**
**Ruiz-Salmeron** **(2011)**	[120]	BM-MNCs	NC	ASO & others	Rutherford 4–6	20	N/A	100–400	IA	12	**ABI**, AR, DR, **TcPO_2_**
**Amann** **(2009) BONMONT-1**	[114]	BM-MNCs	NC	N/A	Rutherford 4–6	12	N/A	1100	IM	13.5	**ABI**, AFS, **PFWD**, **TcPO_2_**
		BM-TNCs				39		3000			
**Walter** **(2011) PROVASA**	[20]	BM-MNCs	RCT	ASO & TAO	Fontaine III–IV	40 (19/21)	Placebo	153	IA	3	ABI, AR, DR, **RPS**, **TcPO_2_**, **UH**
**Li** **(2013)**	[147]	BM-MNCs	RCT	ASO	Fontaine III–IV	58 (29/29)	Placebo	10/mL	IM	6	**ABI**, AFS, AR, DR, **RPS**, **UH**
**Teraa** **(2015) JUVENTAS**	[148]	BM-MNCs	RCT	ASO	Fontaine IIB–IV	160 (81/79)	Placebo	500	IA	6	**ABI**, AR, DR, **TcPO_2_**, UH
**Pignon (2017) BALI**	[149]	BM-MNCs	RCT	ASO	Rutherford 4–5	36 (17/19)	Placebo	1300	IM	12	ABI, AR, RPS, **TcPO2**, UH
**Guo** **(2018)**	[116]	BM-MNCs	NR	TAO	N/A	59 (40/19)	Blank	3500	IM	129.5	**ABI**, **AFS**, **AR**, **RPS**, **TcPO_2_**, **UH**
**Lu** **(2011)**	[150]	BM-MNCs	RCT	ASO	Fontaine IV	21^2^	Blank	930	IM	6	**ABI**, **AR**, **PFWT, RPS**, **TcPO_2_**, **UH**
		BM-MSCs				20^2^		960			
**Dash** **(2009)**	[151]	BM-MSCs	RCT	ASO	N/A	6 (3/3)	Blank	N/A	IM	3	**PFWD**, **UH**
				Buerger		18 (9/9)					
**Gupta** **(2013)**	[152]	BM-MSCs (allogenic)	RCT	ASO & TAO	Rutherford 4–6	20 (10/10)	Placebo	200	IM	6	**ABI**, AR, **RPS**, **UH**
**Szabò** **(2013)**	[153]	Ves-Cell	RCT	N/A	Fontaine III–IV	20 (10/10)	Blank	66.4	IM	3	ABI, AR, DR, PFWD, RPS, TcPO_2_, UH
			NC							22.6	**ABI**, **AFS**, **AR**, **DR**, PFWD, **RPS**, **TcPO_2_**, **UH**
**Raval** **(2014) SCRIPT-CLI**	[154]	CD133+^1^	RCT	ASO	N/A	10 (3/7)	Placebo	50–400	IM	12	AFS, AR, DR
**Lara-Hernandez** **(2010)**	[155]	EPCs^1^	NC	ASO & TAO	Fontaine III–IV	28	N/A	N/A	IM	14.7	**ABI**, **RPS**, **UH**
**Kinoshita** **(2012)**	[156]	CD34+^1^	NC	ASO & Buerger	Rutherford 4–5	17	N/A	0.1/kg (LD) 0.5/kg (MD) 1/kg (HD)	IM	12	**AR**, **DR**, **PFWD**, **RPS**, **TcPO_2_**, UH
**Dong** **(2013)**	[157]	CD34+^1^	NC	ASO, TAO & others	Rutherford 4–5	25	N/A	0.1/kg (LD) 0.5/kg (MD) 1/kg (HD)	IM	6	**ABI**, AR, **DR**, **PFWT**, **RPS**, **TcPO_2_**, **UH**
**Fujita** **(2014)**	[158]	CD34+^1^	NC	ASO & Buerger	Rutherford 4–5	11	N/A	1/kg	IM	12	AR, **PFWD**, **RPS**, **TcPO_2_**
**Powell** **(2012) RESTORE-CLI**	[159]	Ixmyelocel-T	RCT	N/A	N/A	72 (48/24)	Placebo	35–295	IM	12	AFS, AR, DR
**Losordo** **(2015)**	[160]	CD34+^1^	RCT	N/A	Rutherford 4–5	28 (16/12)	Placebo	0.1/kg (LD) 1/kg (HD)	IM	12	ABI, AR, DR, PFWD, UH
**Liotta (2018)**	[161]	BM-MNCs	R	N/A	Rutherford 4–6	17	Blank^5^	50^6^	IM	12	**ABI**, **PFWD**, **RPS**, **TcPO2**, **UH**
		ECEPCs				23		250^6^			
**Fang (2020)**	[162]	PB-MNCs^1^	RCT	TAO	Rutherford 4–5	78	PB-MNC	70, 37^7^	IM	46,6	**ABI**, AFS, **PFWT**, **RPS**, **TcPO2**
		CD34+^1^				82		31, 95^7^			
**Sharma (2021)**	[163]	BM-MNCs	RCT	ASO & others	Fontaine IIC–IV	81 (41/40)	Placebo	71, 51	IA	6	**ABI**, **AR**, PFWD, RPS, **TcPO2**, UH

^1^ Cells mobilized with G-CSF; ^2^ The other limb was used as control, injected with saline serum; ^3^ Each limb was randomized for PB-MNCs/BM-MNCs; ^4^ Same control was used; ^5^ Retrospective; ^6^ Quantity referred to CD14+CD34^low^ cells; ^7^ Quantity referred to CD34+ cells.

## Data Availability

Not applicable.

## Abbreviations

a	Autologous
ABI	Ankle brachial index
aBMCs	Autologous bone marrow cell transplantation
AD	Arteriolar density
ADRCs	Adipose-derived regenerative cells
AFS	Amputation free survival
AFSCs	Amniotic fluid-derived stem cells
AI	Angiographic index
AR	Amputation rate
ASCs	Adipose tissue derived stem cells
ASO	Atherosclerosis obliterans
BM-MNCs	Bone marrow-derived mononuclear cells
BM-MSCs	Bone marrow mesenchymal stem cells
BM-TNCs	Bone marrow total nucleated cells
BFP	Blood flow perfusion
CACs	Circulating angiogenic cells
CBF	Calf blood pressure
CD	Capillary density
CM	Conditioned medium
CLI	Critical limb ischemia
CVDs	Cardiovascular diseases
CVF	Collateral vessel formation
DM	Diabetes mellitus
DR	Death rate
ECFCs	Endothelial colony forming cells
ECs	Endothelial cells
ECEPCs	Enriched circulating endothelial progenitor cells
EPCs	Endothelial progenitor cells
ESC-ECP	Stem cell-derived endothelial cell product
FAL	Femoral artery ligation
FGF1	Fibroblast growth factor 1
FS	Functional score
G-CSF	Granulocyte colony-stimulating factor
HD	High dose
HGF	Hepatocyte growth factor
HIF-1a	Hypoxia-inducible factor 1-alpha
HPCs	Hematopoietic progenitor cells
h	Human
IA	Intraarterial
IC	Intracardiac
IHC	Immunohistochemistry
IM	Intramuscular
IV	Intravenous
LD	Low dose
LDP	Laser Doppler Perfusion
MACs	Myeloid angioenic cells
MD	Medium dose
MMPs	Matrix metalloproteinases
MP	Matrigel plug
MSCs	Mesenchymal stem cells
NC	Non-controlled
NO	Nitric oxide
NR	Non-randomized
PAD	Peripheral arterial disease
PB-MNCs	Peripheral blood mononuclear cells
PFWD	Pain-free walking distance
PRP	Platelet-rich plasma
RCT	Randomized controlled trial
RPS	Rest pain score
SC	Subcutaneous
SMCs	Smooth muscle cells
SMPCs	Smooth muscle progenitor cells
SVF	Stromal vascular fraction
TAO	Thrombo-angiitis obliterans
TcPO_2_	Transcutaneous oxygen pressure
TR	Tissue regeneration
UH	Ulcer healing
VAS	Visual analogue scale
VD	Vessel diameter
VEGF	Vascular endothelial growth factor
VIP	Vascular intersection percentage
VS	Visual Scale
VSEL	Very small embryonic-like stem cells
